# Developing quality indicators for physician-staffed emergency medical services: a consensus process

**DOI:** 10.1186/s13049-017-0362-4

**Published:** 2017-02-15

**Authors:** Helge Haugland, Marius Rehn, Pål Klepstad, Andreas Krüger, The EQUIPE-collaboration group, Gry Elise Albrektsen, Peter Anthony Berlac, Geir Sverre Braut, Per Bredmose, Robert Burman, Brian Burns, Alasdair Corfield, Marta Ebbing, Magnus Hjortdahl, Freddy Lippert, Pål Madsen, Per Oretorp, Leif Rognås, Julian Thompson, Oddvar Uleberg, Janne Virta, Wolfgang G. Voelckel, Ryan Wubben

**Affiliations:** 1 Department of Research and Development, Norwegian Air Ambulance Foundation, Drøbak, Norway; 2 Department of Emergency Medicine and Pre-Hospital Services, St. Olavs Hospital, Trondheim, Norway; 3 Department of Circulation and Medical Imaging, Medical Faculty, NTNU, Norwegian University of Science and Technology, Trondheim, Norway; 4 Department of Health Studies, University of Stavanger, Stavanger, Norway; 5 Division of Emergencies and Critical Care. Department of Anaesthesia, Oslo University Hospital, Oslo, Norway; 6 Department of Anaesthesiology and Intensive Care, St. Olav University Hospital, Trondheim, Norway

**Keywords:** Quality indicators, Physician-staffed emergency medical services, Modified nominal group technique

## Abstract

**Background:**

There is increasing interest for quality measurement in health care services; pre-hospital emergency medical services (EMS) included. However, attempts of measuring the quality of physician-staffed EMS (P-EMS) are scarce. The aim of this study was to develop a set of quality indicators for international P-EMS to allow quality improvement initiatives.

**Methods:**

A four-step modified nominal group technique process (expert panel method) was used.

**Results:**

The expert panel reached consensus on 26 quality indicators for P-EMS. Fifteen quality indicators measure quality of P-EMS responses (response-specific quality indicators), whereas eleven quality indicators measure quality of P-EMS system structures (system-specific quality indicators).

**Discussion:**

When measuring quality, the six quality dimensions defined by The Institute of Medicine should be appraised. We argue that this multidimensional approach to quality measurement seems particularly reasonable for services with a highly heterogenic patient population and complex operational contexts, like P-EMS. The quality indicators in this study were developed to represent a broad and comprehensive approach to quality measurement of P-EMS.

**Conclusions:**

The expert panel successfully developed a set of quality indicators for international P-EMS. The quality indicators should be prospectively tested for feasibility, validity and reliability in clinical datasets. The quality indicators should then allow for adjusted quality measurement across different P-EMS systems.

**Electronic supplementary material:**

The online version of this article (doi:10.1186/s13049-017-0362-4) contains supplementary material, which is available to authorized users.

## Background

The European Resuscitation Council has identified five critical conditions that require immediate pre-hospital management; cardiac arrest, severe respiratory failure, severe trauma, chest pain and stroke. Four of these conditions are among the leading causes of death in the European Union [1]. An observational study on Scandinavian physician-staffed emergency medical services (P-EMS) observed a pre-hospital incidence of severe illness or injury of 25–30 per 10 000 person-years [2]. Many of these conditions benefit from interventions that rapidly correct deranged physiology and improve tissue oxygen delivery [3]. Services delivering pre-hospital critical care remain a critical link in the chain of survival for several frequent and life-threatening conditions.

Pre-hospital emergency care is primarily delivered by paramedics or nurses in ambulance EMS. In addition many health care systems employ P-EMS to respond to selected patients [4–6]. These P-EMS normally use rapid response cars or helicopters depending on distance to the scene and receiving hospital, weather, and the characteristics of each assignment [7]. However, although P-EMS is widely established in many countries little is known about the quality delivered by P-EMS.

The importance of quality measurement in health care is widely recognized [8–11]. Moreover, defining quality indicators (QI) for P-EMS and EMS is identified as a high priority research area [12, 13]. QIs are instrumental to aid clinicians, organizations, health care managers and societies to achieve improvements in health care quality [14]. Further, QIs should integrate the best research evidence with clinical expertise and patient values [15] and allow measurement of health care quality by creating a quantitative basis that indicates performance.

The literature on QIs in pre-hospital critical care is scarce [13, 16] and there is no international agreement on conceptual framework or choice of QIs for P-EMS. Appropriate QIs are needed to identify both high-quality care as well as areas where there is room for improvement in care. The current study describes the development of a comprehensive set of QIs for P-EMS and is a necessary initial step towards quality measurement in this field of health care.

## Methods

### Conceptual framework

For the purpose of this study, we used the framework described by Donabedian, which groups QIs in three broad categories; structure, process or outcome of health care [17, 18]. Structure indicators describe the infrastructure of a health care system, such as competence of the staff, available equipment, deployment and response times. Process indicators evaluate the care provided to the patient, whereas outcome indicators address the change in the patient’s health status as a result of the provided care. Each type of QI will not give a complete description of the quality of care, but rather addresses a component of the care. Thus, different types of QIs should be combined when estimating the quality of a service [14].

**Fig. 1:**
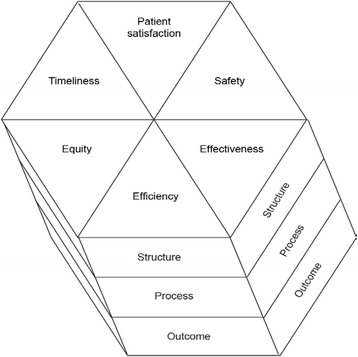
Conceptual framework for multidimensional quality measurement in P-EMS

To identify potential QIs, a widely used method is a combination of a systematic review of current literature and a formal process to obtain expert opinions. In this study, we tasked an expert panel to develop QIs for P-EMS using the modified nominal group technique [19, 20]. We defined P-EMS as a dedicated unit staffed with physicians trained in emergency care exceeding the competency of a general practitioner on call [21]. The QIs should be feasible to collect during the pre-hospital time interval or in the emergency department at hand-over. Further, the QIs should as far as possible cover the six quality dimensions that define high-quality care, stated by the Institute of Medicine [22], and appreciated by the World Health Organization [10]. The six quality dimensions are timeliness, safety, efficiency, equity, effectiveness, and patient-centeredness. An overview of the conceptual framework for this study; using structure-, process- and outcome-indicators to address six established quality dimensions, is depicted in Fig. 1.

### The experts

When developing QIs the expert panel should consist of people considered experts in the appropriate area and who have credibility in the target audience [19]. Clinical expertise is represented by physicians, scientific expertise by researchers and user-expertise by patients. Accordingly, this study’s expert panel consisted of clinicians and researchers from different P-EMSs, but also of stakeholders representing other perspectives in P-EMS. More specifically the 18 members of the expert panel consisted of, three general practitioners, two P-EMS medical directors, a director of a public health institute, a specialist in community medicine, a patient-organization leader and ten physicians working in P-EMS. All panel members were in different ways considered experts in P-EMS or in collaborating services of P-EMS, and practiced in Australia, Austria, Denmark, Finland, Norway, Scotland, United Kingdom and the United States of America. The experts were recruited through PubMed and Google Scholar searches, and via the professional network of the study group. 26 experts were invited by e-mail or telephone. 18 accepted the invitation, two declined and two did not respond. Non-responders were reminded three times by e-mail and three times by telephone.

### The modified nominal group process

In our study, the expert panel developed the QIs through a four-step modified nominal group technique. Stage 1, 2 and 4 were e-mail correspondences. In stage 3, the expert panel gathered for a 2-day consensus meeting in Oslo, Norway.

Stage 1. The members of the expert panel were asked to propose QIs for P-EMS according to the following predefined instructions: A total of 3–10 QIs should be proposed for each of the three categories of QIs; structure, process and outcome. A fourth category (Other indicators) was available for proposing QIs difficult to fit into the Structure-Process-Outcome - system. All proposed QIs should be possible to obtain during the pre-hospital time interval. The experts were asked to consider both evidence base and feasibility of data-collection when proposing QIs. However, it was not required that the proposed QIs could be extracted from existing databases in P-EMS.

The panel members returned their proposals to the project group pr. e-mail in a predesigned Excel spreadsheet (Microsoft Office 2013, Microsoft Inc., USA). QIs with identical meaning were merged. No proposed QIs were deleted. Further, the QIs within each category (structure, process and outcome) were ranked according to the number of experts who had included each QI in their proposal.

Stage 2. The experts were asked to use the revised spreadsheet to rank the ten most important QIs in each of the three categories (structure, process and outcome). In each category, the quality indicator ranked in first place was given a point value of 10, second of 9, third of eight and so forth, until the tenth place that was given one point. The point values from all panel members were added, and quality indicators with no ranking were removed from the list. The list with the remaining quality indicators, prioritized according to achieved point value, formed the basis for the consensus meeting.

Stage 3. The expert panel gathered for a 2-day consensus meeting in Oslo, Norway. A moderator (MR) led the experts through discussions on the QIs in the spreadsheet developed in stage 2. The experts decided which QIs should be included in the final set. Further, preliminary definitions and limitations were defined. All debates and discussions were plenary.

Stage 4. Based on the results from the consensus meeting, the project group prepared a document with the selected QIs, including definitions. This document was submitted to the panel members for comments. At this stage, no additional QIs were accepted. However, minor changes pertaining to definitions were allowed.

Consensus was defined as agreement on the proposed QIs during the meeting among the attending experts.

## Results

The 18 experts proposed and ranked QIs in stage 1 and 2 (one expert did not submit rankings in stage 2). In stage 1, 358 QIs were proposed by the expert panel. After merging, 179 QIs entered stage 2. At stage 2, 45 QIs obtained 0 points and were excluded, leaving 134 indicators to be discussed at the consensus meeting. Thirteen experts attended the consensus meeting. During the consensus meeting the expert panel recommended the QIs from stage 2 to be classified into two different categories for clarity; response-specific QIs and system-specific QIs. The former is data from the pre-hospital time interval, measuring quality on the response level, and should be feasible to collect from any P-EMS response by the P-EMS physician. The latter should be administrative data describing fixed system characteristics, and should be registered once a year for services using the set of quality indicators continuously or for study purposes. The expert panel argued that the combined information from response- and system-specific QIs allows for a more thorough quality measurement than exclusively relying on response-specific QIs.

Consensus was reached on 15 response- and 11 system-specific QIs (Tables 1 and 2). More specific definitions for each QI are given in the explanation and elaboration document (Additional file 1). The expert panel allowed the project group to finalize the definitions of the indicators and propose them to all 18 experts in stage 4, where the final result was agreed upon. The QIs were allocated into one of the six quality dimensions as defined by the Institute of Medicine. All six quality dimensions were covered by the QIs, and both structure-, process- and outcome-indicators were represented. An overview of the distribution of the QIs is presented in Table 3.

**Table 1:** Response-specific quality indicators for physician-staffed emergency medical services

#	Quality indicator	Type of quality indicator	Quality dimension
1	Was the P-EMS unit *able* to respond immediately to the actual response?	Structure	Timeliness
2	What is the time interval from the dispatch center receives the alarm call until P-EMS unit arrives at the patient?	Structure	Timeliness
3	What is the time interval from P-EMS unit arrives at the patient until transportation of patient is initiated?	Process	Timeliness
4	What is the time interval from the P-EMS unit received the alarm call until the patient was delivered at the preferred destination?	Process	Timeliness
5	Did the patient arrive hospital alive?	Outcome	Timeliness
6	Was the P-EMS response debriefed?	Process	Safety
7	Did you experience any adverse events during the P-EMS response?	Process	Safety
8	Are all defined key variables measured and documented in the patient chart?	Process	Efficiency
9	Did the service have a guideline for the medical problem encountered in the response?	Structure	Equity
10	Was a physician and/or a paramedic from P-EMS involved in deciding if the P-EMS unit should be dispatched to the particular job or not?	Process	Equity
11	Without the assistance of the P-EMS unit: Do you consider that the level of competence on scene was sufficient to give the patient appropriate care?	Process	Equity
12	Did P-EMS provide advanced treatment in the actual response?	Process	Effectiveness
13	Did the logistical contribution by P-EMS give the patient a significant better service than the existing alternative?	Process	Effectiveness
14	Was the patient enrolled in a scientific study involving the pre-hospital care?	Structure	Effectiveness
15	Did you ensure that the relatives’ needs were addressed; either by P-EMS or by collaborating services?	Process	Patient-centeredness

**Table 2:** System-specific quality indicators for physician-staffed emergency medical services

#	Quality indicator	Type of quality indicator	Quality dimension
16	Is the dispatch center staffed 24/7 by specially trained pre-hospital physician?	Structure	Effectiveness
17	What is the number of P-EMS units per 100 000 inhabitants in the service area?	Structure	Equity
18	What is the number of P-EMS units per km^2^ in the area covered by the service?	Structure	Equity
19	Does the service regularly perform interfacility transports coordinated by a dispatch centre?	Structure	Effectiveness
20	What level of regular in-hospital service do the P-EMS doctors practice in addition to their pre-hospital work?	Structure	Effectiveness
21	Proportion of P-EMS doctors with achieved speciality in: 1; anesthesiology 2; emergency medicine 3; other specialities.	Structure	Effectiveness
22	Proportion of P-EMS doctors who have attended and passed formalized training in major incident management.	Structure	Efficiency
23	Proportion of P-EMS doctors’ assistants with the following qualification: Paramedic or nurse with supplemental regular training in assisting during induction of general anesthesia and/or formal education in anesthesia or intensive care.	Structure	Safety
24	Does the P-EMS service collect data pertaining to patient satisfaction?	Structure	Patient- centeredness
25	What is the number of documented complaints from patients, relatives or receiving hospitals per total number of P-EMS events (ratio)?	Outcome	Patient- centeredness
26	Does it exist a system for registration and reviewing of adverse events, critical incidents and educational events in the service?	Structure	Safety

**Table 3:** Classification of quality indicators from the consensus process

	Timeliness	Safety	Efficiency	Equity	Effectiveness	Patient-centeredness	Number	Percent
Structure	0	2	1	2	6	1	12	46,2
Process	4	1	1	2	2	1	11	42,3
Outcome	1	1	0	0	0	1	3	11,5
n	5	4	2	4	8	3	26	
%	19,2	15,4	7,7	15,4	30,8	11,5		

## Discussion

This paper presents a set of potential QIs for quality measurement of P-EMS. Using a modified nominal group technique an international expert panel achieved consensus on these QIs that describe six quality dimensions and include structure-, process- and outcome-indicators.

Quality measurement of pre-hospital services has been identified as a high-priority research area and pivotal to achieve improvement in care [12, 13, 23, 24]. However, identifying valid quality indicators that are feasible to collect in the operational context of pre-hospital services has been a challenge [25]. We deliberately asked the experts to propose quality indicators themselves, not simply selecting from a pre-defined list. The rationale behind this was to make this process as open as possible in order to achieve a broad selection of proposals. The multidisciplinary composition of the expert panel was partly to facilitate this broad approach.

A premise for this study is that the principles for quality measurement in health care also applies to P-EMS. P-EMS is the practice of medicine outside hospitals, and we find it reasonable to accept this premise. The six core characteristics of quality depicted in Fig. 1 were defined by Institute of Medicine, naming them dimensions of quality [22]. Each of these is distinct and no one is defined more important than the others. When measuring quality, all six quality dimensions should be appraised. We argue that this multidimensional approach to quality measurement seems particularly reasonable for services with a highly heterogenic patient population, like P-EMS. Patients cared for by P-EMS differ a lot: Neonates vs. elderly patients, medical vs. surgical diagnoses, patients rescued from open water vs. Intensive Care Unit transferals [2, 5]. What is considered high quality care for each patient will be context-specific. With this complexity of cases, treatments and operational contexts, we argue that adequate quality measurement of P-EMS should be multidimensional.

### Quality dimensions

Timely care is about reducing needless and potentially harmful delays before the patient receives specialized care from the P-EMS. Traditionally, attempts on quality measurement of pre-hospital services, have been limited to data on time-variables corresponding the quality dimension *“timeliness*” [13, 26]. Studies of EMS have shown that response time affects outcome only for a small group of patients [27, 28]. Moreover, time variables describe the logistics, but not the provided care. Response time of P-EMS is measured in QI 2 “Time to arrival of P-EMS” and is indeed important for some time critical conditions such as cardiac arrest and major trauma [29]. However, the importance of short response times cannot be generalized to all emergency responses [30]. In selected situations, too much emphasis on timeliness is misleading in respect of what really represents quality for the patient. In the United Kingdom paramedics criticized the use of a time target structure measure (eight-minute response time for 75% of category A or emergency calls) as the main performance indicator in EMS. They argued this QI was “too simplistic and narrow” and that it could also increase risk for patients and ambulance crews [31]. An example may illustrate the limitation of time-variables as the sole QIs in P-EMS: Performing an ultra-sound scan of the traumatized patient may prolong the time on scene slightly. However, the examination can result in changes in treatment or triage [32], hence making the extra time spent on scene well worth.

The quality dimension *“safety”* focus on safety issues related to P-EMS responses for patient, EMS-staff or others. The safety issues can be medical, technical or operational. P-EMS operates rapid response cars and helicopters, all activities associated with operative risks for patients, bystanders and crew [33]. Additionally, P-EMS care for severely injured or ill patients without access to safety initiatives as seen in hospitals e.g. senior assistance or access to patient history. Moreover, the pre-hospital environment can be associated with hazards like extreme temperatures, traffic and difficult access requiring application of rescue techniques [34].

The quality dimension “*efficiency”* is about avoiding medical waste; including waste of use of P-EMS personal, equipment and energy. Advanced major incident management reduce over-triage and is an example of how to prevent waste of resources [35]. This issue is covered in QI 22, which measures the proportion of P-EMS doctors who have completed a major incident management program.

*“Equity”* is about ensuring that quality of care is provided equally regardless of the patient’s gender, ethnicity, geographic location and socioeconomic status. P-EMS contributes to equitable care by reducing transportation times (when using a helicopter) and by bringing the hospital competencies to the pre-hospital environment. This role of P-EMS can also be defined a governmental objective [36] as an initiative to give people living in scattered spread populations specialized care within due time. Thus, a more equitable access to centralized medical treatments like neurosurgery and invasive cardiology can be provided. The expert panel argued that the involvement of a physician or a paramedic from P-EMS in the dispatch decision would secure the most correct use of P-EMS, thus contributing to equitable care. This is addressed in QI 9 «P-EMS involvement in dispatch decision».

*“Effectiveness”* is about ensuring that provided treatment is evidence-based. Care proven effective should be provided, thereby preventing undertreatment. Similarly, care proven ineffective should not be provided, thereby preventing overtreatment. There is some evidence that the use of physicians in EMS for selected patient groups, improve outcome or proxy outcomes such as physiological variables [1, 37]. However, the current documentation on the impact of P-EMS initiatives is controversial and, therefore, effectiveness QIs are difficult to derive from the literature. The expert panel combined existing evidence with the experience and considerations of all panel members. One of the resulting QIs, QI 12 “Advanced Treatment”, addresses care considered indicated, but not feasible without the competence of P-EMS. Please note that withholding unethical or unnecessary treatment by the P-EMS physician also was defined as “advanced treatment” by the expert panel. Thus, critical decision making as illustrated for pre-hospital advanced airway management by Rognås et al.[38], is recognized as a part of quality care.

*“Patient-centeredness”* is about ensuring that care is responsive to individual needs. Although most stakeholders and clinicians in P-EMS presumably put the patient in the center of the care, the study group wanted to secure that the patients were represented in the expert panel. Therefore, a leading representative from a major patient organization was invited to join the expert panel. Developing quality indicators for this quality dimension in P-EMS is challenging, primarily because many of the patients cared for by the service are unconscious or at least not capable of expressing their own needs in their usual manner. This can be due to the clinical condition itself, stressful situation or pharmacological interventions. The needs of the patient’s family, however, can be expressed more easily. Moreover, the term “patient-centeredness” has been argued expanded to “patient- and family-centeredness” [39]. Patient- and family-centered care is based on the beneficial partnership between patient, family and health care workers, and it can be applied to patients in all ages and in any health care setting [39, 40]. As a surrogate for measuring the patient’s needs, the needs of the patient’s relatives could be addressed, as defined in QI 15 “Care for relatives”. This QI addresses the relatives’ needs, including the need for practical and emotional assistance.

### Types of quality indicators

J. Mainz has reviewed the strengths of structure-, process- and outcome-indicators [14]. Structure indicators are found most useful when they predict variations in processes or outcomes of care. Process indicators are particularly useful when coping with short time frames, low volume providers and when tools to adjust or stratify for patient related factors are difficult to apply. Comparison of process indicators are generally easier to interpret and more sensitive to small differences than comparison of outcomes data. Based on these characteristics, we consider process indicators particularly suitable for continuous quality measurement of P-EMS. Although necessary to get information about a patient’s final outcome, long-term outcome indicators appear less feasible for measuring the isolated quality of P-EMS. From a patient is admitted to hospital by P-EMS until a long-term outcome is measured, the patient has received care from numerous units, each potentially influencing outcome [41]. Unless performing risk adjustment and outcome measurement for each of these care intervals, it will problematic to use long term outcome measures as indicators of the isolated quality of P-EMS. Instead, quality indicators from the pre-hospital care interval should be developed for this purpose [23]. The Institute of Medicine has stated that «quality of care is the degree to which health services for individuals and populations increase the likelihood of desired health outcomes and are consistent with current professional knowledge» [42]. This definition of quality is a reminder that good quality is not identical to good outcomes. Despite excellent health care is provided, outcome for patients can be poor. Opposite, patients receiving poor quality health care can have good outcome.

### Strengths and limitations

Using the professional network of the study group for recruitment of panel members, may have limitations: Colleagues that share our own professional interests may have been easier to identify and invite, than those with other views and mindsets. This practice can possibly lead to an imbalance in the composition of the expert panel. Although the expert panel reflected the inter-disciplinary nature of EMS, we recognize that we did not include a representative from an Emergency Medical Communication Central (EMCC). There was a trade-off between a manageable number of experts and the need for an inter-disciplinary composition of the expert panel. Consensus methodology literature describe an optimal group size of eight to twelve members [43]. Our efforts in making the panel sufficiently inter-disciplinary resulted in a group size of 18 experts. However, due to a rigorous time schedule throughout the process, the slightly larger expert panel did not lead to any unnecessary delay.

Eight nations were represented in the expert panel; all from developed countries and the majority from Scandinavia. Therefore, we recognize that other areas may have other QIs which should be implemented locally. However, P-EMS as a service is usually only delivered in d eveloped countries. Hence, for these services the nationalities included should be representative.

In the consensus process we used a system of ranking and scoring to identify the QIs supported by the most experts in the panel. There are different methods to prioritize proposals and obtain consensus, and no method is considered clearly superior to the others [44]. At the consensus meeting, any proposal was omitted if vigorously opposed by one or more of the participants.

The use of a Likert scale is another recognized method for defining the level of consensus. Likert scores are used for QI selection in several studies, including a recent Danish study selecting QIs for hospital-based emergency care [45, 46]. Whether the use of a Likert scale would have improved our consensus process remains unclear. Moreover, it is methodological important to prevent that verbally skilled panel members dominate the consensus process. This issue also relates to “strong” personalities or experts enjoying a higher reputation than the other panel members [19]. Therefore, proposals and rankings in stage 1 and 2 were anonymous.

The value of this study is the development of multidimensional quality indicators for P-EMS. This represents a starting point for future studies on measuring and improving quality of P-EMS. The necessary next step should be to test the feasibility and validity of the QIs in a sample of P-EMSs. Thus, a more final set of QIs for P-EMS can be developed.

## Conclusion

Using a modified nominal group technique, an international expert panel reached consensus on 15 response specific and 11 system specific quality indicators for P-EMS. All six quality dimensions stated by Institute of Medicine are covered, and the quality indicators represent structure, process and outcome indicators. This 26 quality indicators large set is developed to represent a broad and comprehensive approach to quality measurement in international P-EMS, allowing future quality measurement comparable across different P-EMS systems.

## Additional file

Additional file 1: “Definition catalogue from the EQUIPE-project”. This explanation and elaboration document contains the definitions of all quality indicators, as well as explanation of the response alternatives where necessary. (ZIP 134 kb)

## Abbreviations

EMCC: Emergency Medical Communication Central
EMS: Emergency medical service
P-EMS: Physician-staffed emergency medical service
QI: Quality indicator
