# Supplementary material for: Developing quality indicators for physician-staffed emergency medical services: a consensus process
Source: Scand J Trauma Resusc Emerg Med. 2017 Feb 15;25:14. doi: 10.1186/s13049-017-0362-4 (PMC5311851; doi:10.1186/s13049-017-0362-4)
Supplement: Additional file 1: — “Definition catalogue from the EQUIPE-project”. This explanation and elaboration document contains the definitions of all quality indicators, as well as explanation of the response alternatives where necessary. (ZIP 134 kb) [file 13049_2017_362_MOESM1_ESM.zip › Additional file 1.docx]

Definition catalogue from

the EQUIPE-project

Table of contents

[Contributors 3](#_Toc464127957)

[Authors 3](#_Toc464127958)

[Expert panel (collaborators) 3](#_Toc464127959)

[Abbreviations 4](#_Toc464127960)

[Background and aim of the EQUIPE consensus process 5](#_Toc464127961)

[The consensus meeting; mandate and content 5](#_Toc464127962)

[Response-specific quality indicators 6](#_Toc464127963)

[Timeliness 6](#_Toc464127964)

[QI 1 Availability 6](#_Toc464127965)

[QI 2 Time to arrival of P-EMS 7](#_Toc464127966)

[QI 3 On-scene time 8](#_Toc464127967)

[QI 4 Time to definitive care pathway: 9](#_Toc464127968)

[QI 5 Survival to hospital admission: 10](#_Toc464127969)

[Safety: 11](#_Toc464127970)

[QI 6 Debriefing: 11](#_Toc464127971)

[QI 7 Adverse events: 12](#_Toc464127972)

[Efficiency: 13](#_Toc464127973)

[QI 8 Data compliance and completeness: 13](#_Toc464127974)

[Equity: 15](#_Toc464127975)

[QI 9 P-EMS-involvement in dispatch decision: 15](#_Toc464127976)

[QI 10 Need for P-EMS in relation to level of competence on scene: 16](#_Toc464127977)

[Effectiveness: 17](#_Toc464127978)

[QI 11 Guidelines: 17](#_Toc464127979)

[QI 12 Advanced treatment: 18](#_Toc464127980)

[QI 13 Logistics: 19](#_Toc464127981)

[QI 14 Research: 20](#_Toc464127982)

[Patient-centeredness: 21](#_Toc464127983)

[QI 15 Care for relatives: 21](#_Toc464127984)

[System-specific quality indicators 22](#_Toc464127985)

[QI 16 Physician-staffed Emergency Medical Communication Central (EMCC): 22](#_Toc464127986)

[QI 17 P-EMS units per population: 23](#_Toc464127987)

[QI 18 P-EMS units per km²: 24](#_Toc464127988)

[QI 19 Transfer and retrieval service: 25](#_Toc464127989)

[QI 20 Type of in-hospital service: 26](#_Toc464127990)

[QI 21 Proportion of specialists: 27](#_Toc464127991)

[QI 22 Major incident medical management: 28](#_Toc464127992)

[QI 23 Qualification of P-EMS doctor`s assistant: 29](#_Toc464127993)

[QI 24 Patient satisfaction: 30](#_Toc464127994)

[QI 25 Complaints: 31](#_Toc464127995)

[QI 26 Clinical governance: 32](#_Toc464127996)

[Key variables: 33](#_Toc464127997)

[Data variable no. 1: 33](#_Toc464127998)

[Data variable no. 2: 34](#_Toc464127999)

[Data variable no. 3: 35](#_Toc464128000)

[Data variable no. 4: 36](#_Toc464128001)

[Data variable no. 5: (for trauma patients only) 37](#_Toc464128002)

[Data variable no 6: 38](#_Toc464128003)

[Data variable no. 7: 39](#_Toc464128004)

[Data variable no. 8: 40](#_Toc464128005)

[Data variable no. 9: 41](#_Toc464128006)

[Data variable no. 10: 42](#_Toc464128007)

[Data variable no. 11: 43](#_Toc464128008)

[Data variable no. 12: 44](#_Toc464128009)

[Data variable no. 13: 45](#_Toc464128010)

[Data variable no. 14: 46](#_Toc464128011)

[Data variable no. 15: 47](#_Toc464128012)

[Data variable no. 16: 48](#_Toc464128013)

[Data variable no. 17: 49](#_Toc464128014)

[Data variable no. 18: 50](#_Toc464128015)

[Data variable no. 19: 51](#_Toc464128016)

[Data variable no. 20: (only for patients who are intubated or have a supraglottic device) 52](#_Toc464128017)

[Data variable no. 21: (only for patients who are intubated or have a supraglottic device) 53](#_Toc464128018)

[References 54](#_Toc464128019)

# Contributors

## Authors

**Helge Haugland**

**Marius Rehn**

**Pål Klepstad**

**Andreas Krüger**

## Expert panel (collaborators)

**Dr. Gry Elise Albrektsen**

General practitioner, Florø, Norway.

**Dr. Peter Anthony Berlac, MHM**

Medical Director, Emergency Medical Services Copenhagen.

Consultant Anaesthesiologist and Prehospital Emergency Physician.

**Dr. Geir Sverre Braut**

Professor, University of Stavanger, Norway.

Former Deputy Director General, Norwegian Board of Health Supervision.

Specialist in community medicine.

**Dr. Per Bredmose**

Consultant in Prehospital Care and Retrieval Medicine, Oslo, Norway. Director of Training.

Consultant Anaesthesiologist.

**Dr. Robert Burman, PhD**

General practitioner and Consultant, Emergency Ward, Kristiansand, Norway.

Researcher, National Centre for Emergency Primary Health Care.

**Dr. Brian Burns, Msc**

Ass. Professor of Emergency Medicine, University of Sydney.

Prehospital & Retrieval Specialist, Sydney HEMS. Ambulance Service of NSW Director of Trauma.

**Dr. Alasdair Corfield**

Consultant in Emergency & Retrieval Medicine

Clinical Director EM (Clyde)

Honorary Clinical Associate Professor, University of Glasgow, Scotland.

**Dr. Marta Ebbing, PhD**

Department Director, Norwegian Institute of Public Health.

Specialist in Cardiology.

**Dr. Magnus Hjortdahl**

General practitioner, Alta, Norway.

**Dr. Freddy Lippert**

CEO Emergency Medical Services, Copenhagen.

Associate Professor, University of Copenhagen.

**Dr. Pål Madsen**

Medical advisor at Luftambulansetjenesten ANS

(Principal organization for norwegian air ambulance services)

**Per Oretorp**

Ass. secretary general, National association for the Traumatically Injured, Norway.

**Dr. Leif Rognås, PhD**

Lead Clinician at the Pre-hospital Critical Care Service. HEMS physician and consultant anaesthetist.

**Dr. Julian Thompson**

Consultant Anaesthesiologist og Deputy Editor Scand J Trauma Emerg Med. England.

**Dr. Oddvar Uleberg**

Consultant in Anaesthesiology and Pre-hospital Critical Care, Trondheim, Norway.

**Dr. Janne Virta**

Consultant Anaesthesiologist, HEMS-doctor. Finland.

**A.o. Univ.‐Prof. Dr. Wolfgang G. Voelckel, M.Sc.**

Department of Anesthesiology and Critical Care Medicine, AUVA Trauma Center, Salzburg, Austria. Medical Director, Christophorus Flugrettung, Vienna, Austria.

**Dr. Ryan Wubben**

Clinical Associate Professor, Department of Emergency Medicine, University of Wisconsin School of Medicine and Public Health, USA.
Medical Director and Flight Physician, UW Med Flight.

# Abbreviations

P-EMS = Physician-staffed emergency medical services

QI = Quality indicator

EMD = Emergency Medical Dispatch

AMI = Acute Myocardial Infarction

SOP = Standard Operating Procedures

EED project = European Emergency Data Project

CPR = Cardio-pulmonary resuscitation

# Background and aim of the EQUIPE consensus process

**Background.** The focus on quality in health care is increasing. There is an interest in measuring, assuring and improving quality. Scientific journals and high-impact organizations are among the initiators of this interest (1-4).

One tool for measuring quality in health care are quality indicators. By creating a quantitative basis, quality indicators tell us how the health system is performing, as well as aid clinicians, organizations and planners to achieve improvement in care. Quality indicators should be evidence-based, or-when scientific evidence is lacking- determined by an expert panel through a consensus process (5).

Estimating the quality of physician-staffed emergency medical services (P- EMS) is necessary in order to achieve appropriate governance, quality assurance and even quality improvement of the service. In Norway, no quality indicators are registered systematically or continuously in physician-staffed EMS, and to the best of our knowledge, this is the situation in many other countries as well.

**Aim.** To establish quality indicators for physician-staffed emergency medical services.

**Method.** The establishment of quality indicators for P-EMS was conducted through a four-step modified nominal group technique with an expert panel consisting of clinicians, researchers and other stakeholders considered experts in different fields of P-EMS, or fields strongly related to P-EMS. We have now finished the consensus meeting (third step), and are proceeding with the fourth step - the summary final e-mail round.

**Significance.** First international study to systematically develop and agree upon quality indicators for P-EMS.

# The consensus meeting; mandate and content

Prior to the consensus meeting, the members of the expert panel had proposed a total of 178 quality indicators. After the ranking, 45 quality indicators were removed, as result of receiving 0 points in the ranking. The expert panel`s mandate at the consensus-meeting, was to agree upon 1-4 quality indicators for each quality dimension, as stated by the Institute of Medicine and also appreciated by the World Health Organization (3, 6). The expert panel reached consensus on 17 quality indicators. Additionally, the members of the panel agreed that a list of condition specific measurements is necessary, and that this list will be developed during this 4^th^ and last round of the study. Finally, the expert panel emphasized the need for system-specific variables when comparing different services. Agreement on a list of such system-specific variables will be an additional aim in this closing round of the consensus process.

# Response-specific quality indicators

## Timeliness

The expert panel agreed on the following quality indicators for the quality dimension of timeliness:

### QI 1 Availability

**Definition of QI:** Was the P-EMS-unit *able* to respond immediately to the actual response?

- Yes
- No

**Why this QI is important:** P-EMS should be a reliable service to the population, not limited by service hours, and resilient against austere weather conditions or aviation technical issues.

**Source of information:** P-EMS and Emergency Medical Communication Central (EMCC).

**Comments:** The availability of the service must not affect the safety of the service.

The term “immediately” means: An immediate response compared to normal conditions/normal reaction time.

For this QI to be used optimally, the P-EMS-doctor must be notified of every response considered indicated for the P-EMS unit by the EMCC. This has to be done also in situations where it is obvious that the P-EMS unit cannot dispatch (unavailability due to technical reasons, service hours, weather, concurrency conflicts etc.) When it is obvious for the EMCC that the P-EMS-unit cannot dispatch, the notification of the P-EMS unit can wait until timing is considered appropriate. For services in which the P-EMS doctor him-/herself decides whether to accept the response, the answer on the QI is “Yes” if the P-EMS unit is *able* to respond to the response, but refrains from doing so because of lacking indication as judged by the doctor.

**Other quality dimension covered by this QI:** Equity. Effectiveness.

**Type of quality indicator:** Structure

### QI 2 Time to arrival of P-EMS

**Definition of QI:** What is the time interval from the EMCC receives the alarm call until P-EMS unit arrives at the patient?

- Time interval: in minutes.

**Why this QI is important:** The patient is requesting medical help. It is considered good quality to reduce waiting and harmful delays. A core task for P-EMS is to bring specialist competence to the pre-hospital environment, and the doctor is the core resource in this task.

**Source of information:** Medical reports, patient data management system, monitoring and dispatch data.

**Comments**: In non P-EMS systems, the patient will first be seen (at some point) by a specialist in anesthesiology/critical care/emergency medicine when he/she arrives in hospital. By bringing the hospital competence to the pre-hospital patient, critical care can start at an earlier stage. This is relevant for several medical and surgical conditions. Moreover, bringing specialist competence to the pre-hospital phase of care contributes to equity of access to health care for those living in remote areas.

**Other quality dimension covered by this QI:** Equity.

**Type of quality indicator:** Structure.

### QI 3 On-scene time

**Definition of QI:** What is the time interval from P-EMS unit arrives at the patient, until transportation of patient is initiated?

- Time interval: in minutes.

**Why this QI is important:** On-scene time is recommended minimized for numerous conditions in order to better outcome. However, on-scene time has to be stratified to the actual condition of the patient.

**Source of information:** Medical reports, patient data management system, monitoring and dispatch data.

**Comments**: We choose to define the starting point of this time interval as the time when the P-EMS unit arrives at the patient rather than when the P-EMS unit stops at scene. This is because some patients may be located relatively far away from where the P-EMS unit has to stop/land. Further, the end point of this time interval is the time when transportation of patient is initiated, meaning when the patient *leaves* the scene; regardless of how this is done (helicopter, ground ambulance or by other means).

**Other quality dimension covered by this QI:** None.

**Type of quality indicator:** Process.

### QI 4 Time to definitive care pathway:

**Definition of QI:** What is the time interval from the P-EMS unit received the alarm call until the patient was delivered at the preferred destination as defined by local protocols or by the P-EMS physician her-/himself when a local protocol for this does not exist?

- Time interval: in minutes.
- Patient NOT delivered at the preferred destination.

The expert panel has also defined P-EMS delivered thrombolysis for AMI-patients as definitive treatment, and thus, this time interval (Time to definitive care pathway) will be closed pre-hospital when thrombolytic treatment is delivered.

**Why this QI is important:** Time consumption is critical for conditions like myocardial infarction, ischemic stroke, major traumas etc.

**Source of information:** Medical reports, monitoring and dispatch data.

**Comments**: *Important remark; this QI is for primary missions only.*

This time interval should be closed when the patient is handed over to the responsible staff at the receiving destination; NOT when the helicopter is landing on the hospital`s helideck, for instance.

The preferred destination would often be a hospital. However, it could also be an ambulance, a GP office or even the scene itself in “treat and leave” decisions.

P-EMS physicians would probably not deliberately deliver a patient at the non-preferred destination. Nevertheless, this can occur because of weather conditions, technical problems, concurrency issues, the patient`s condition etc.

P-EMS services are encouraged to define time targets in condition-specific guidelines where appropriate; like time to PCI, time to thrombolysis center for Stroke-patients etc. However, local circumstances differ a lot, and guidelines defining both specific time targets and definitive care might change in the future. Thus; defining the QI as above, is considered more appropriate by the expert panel.

**Other quality dimension covered by this QI:** Effectiveness. Equity.

**Type of quality indicator:** Process.

### QI 5 Survival to hospital admission:

**Definition of QI:** Did the patient arrive hospital alive? Survival to hospital admission (overall; including all patients regardless of clinical condition).

- Yes.
- No.
- Unknown (e.g. When the patient is escorted to hospital by others than P-EMS.)
- Patient arriving hospital under on-going CPR.

**Why this QI is important:** Discrete episodes where rapid response and appropriate care can have an impact that can be isolated to P-EMS initiatives.

**Source of information:** Medical reports, patient data management system.

**Comments**:

**Other quality dimension covered by this QI:** Safety.

**Type of quality indicator:** Outcome.

## Safety:

### QI 6 Debriefing:

**Definition of QI:** Was the P-EMS response debriefed?

- Yes, formal debriefing using a check list.
- Yes, formal debriefing, but without using a check list.
- Yes, informal debriefing.
- No.

As *formal debriefing* we understand: getting the whole P-EMS-crew together for a few minutes after the P-EMS response to discuss in a non-threatening manner what the team did right and to identify those areas where the team needs to improve.

As *informal debriefing*, we understand: the unstructured discussion of only some aspects of the P-EMS response.

“Check list” refers to a check list for debriefing.

**Why this QI is important:** Operational debriefing by the crew can reveal issues pertaining to potential or actual safety problems; both medical and operational. The safety issues might be concerning the patient and/or the P-EMS crew. Furthermore, debriefing can identify where there is room for improvement in terms of cooperation and communication, both within the P-EMS team and with partners like dispatch central, EMS and others.

**Source of information:** Mission chart.

**Comments**:

**Other quality dimension covered by this QI:** None.

**Type of quality indicator:** Process.

### QI 7 Adverse events:

**Definition of QI:**

Did you experience any adverse events during the P-EMS response?

- Yes.
- No.

If “Yes”:

- Characterize the adverse event as:
  - Medical (unexpected death, medication errors, failure of medical (technical) equipment, injuries to patient, staff or others as result of the P-EMS response, etc.)
  - Technical (failure related to the aircraft, the rapid response car, etc.)
  - Operational (logistics, coordination, etc.)
- Did you report the adverse event formally according to existing procedures?
  - Yes.
  - No.

**Why this QI is important:** Learning from adverse events is a prerequisite for improvement. It is important that adverse events are reported, so that colleagues and management can learn from the event.

**Source of information:** Responsible physician. Medical reports, monitoring and dispatch data.

**Comments**: An adverse event is defined as “An untoward, undesirable, and usually unanticipated event, such as death of a patient, an employee, or a visitor in a health care organization. Incidents such as patient falls or improper administration of medications are also considered adverse events even if there is no permanent effect on the patient.”(7)

With the formal reporting of an adverse event, we understand a written report or a completed form, either electronically or by paper.

**Other quality dimension covered by this QI:** None.

**Type of quality indicator:** Outcome.

## Efficiency:

### QI 8 Data compliance and completeness:

**Definition of QI:** Are all defined key variables measured and documented in the patient chart?

- Yes.
- No.

**Why this QI is important:** Reliable and comprehensive data acquisition and documentation is the key requirement for quality assessment and system evaluation. Audit, clinical governance, service development and research all require the analysis of accurate data. Furthermore, complete and compliant documentation is necessary to capture the changes in the patient`s clinical condition. Changes can be post-interventional, a result of the underlying disease or a combination of these.

**Source of information:** Medical reports, monitoring data and dispatch data.

**Comments**: The key variables in the patient chart should be common for participating services. If each service should define their own key variables, this will make benchmarking problematic. As key variables the expert panel defines EtCO2 (first and last) as well as the “patient descriptors” (variables 14-25) in the template for documenting and reporting in P-EMS, by Krüger et al. (8). These variables are developed through an international four-step consensus process, and are designed for physician-staffed EMS.

The key variables are described as key variables 1-21 in this document.

**Other quality dimension covered by this QI:** None.

**Type of quality indicator:** Process.

## Equity:

### QI 9 P-EMS-involvement in dispatch decision:

**Definition of QI:** Was a physician and/or a paramedic from P-EMS involved in deciding if the P-EMS unit should be dispatched to the particular job or not?

- Yes; physician/paramedic from P-EMS currently on call at the P-EMS unit
- Yes; physician/paramedic from P-EMS on call in the EMCC
- Yes; physician/paramedic from P-EMS, but not currently on call at the P-EMS unit or the EMCC
- Unknown
- No

**Why this QI is important:** High service-specific competence secures correct use of the P-EMS unit, thus contributing to equitable use of a limited resource.

**Source of information:** Responsible physician, EMCC.

**Comments**: In services where the acceptance or refusal of a response is at the P-EMS doctors discretion, the P-EMS doctor has been involved in the dispatch decision as long as he/she accepts to be dispatched.

If a response is aborted by the P-EMS doctor en route because of new information etc., this is irrelevant for the content of this question. The question only refers to the decision to dispatch in the first place.

If the EMCC is staffed 24/7 by a pre-hospital trained physician, a secondary benefit might be the use of this physician for major incident command and day-to-day backup/supervision of ambulances (and P-EMS).

**Other quality dimension covered by this QI:** Effectiveness, efficiency .

**Type of quality indicator:** Process.

### QI 10 Need for P-EMS in relation to level of competence on scene:

**Definition of QI:** Without the assistance of the P-EMS-unit: Do you consider that the level of competence on scene was sufficient to give the patient appropriate care?

- Yes
- Probably
- Probably not
- No

Except from the P-EMS-unit: What was the highest level of care at the scene?

- Another P-EMS physician
- General practitioner
- Emergency Medical Service (ambulance)
- Other

**Why this QI is important:** This QI can illustrate when the added competence of P-EMS is needed to provide appropriate care. The P-EMS` ability of decision making will be one of the issues covered by this QI.

There might be situations where there is no ordinary EMS unit available or the competence on scene is not high enough to provide appropriate care. These scenarios may have serious consequences, particularly for patients located far from a hospital. The added competence of P-EMS may contribute to that these patients receive equitable care compared to patients located in more central areas with higher pre-hospital competence and shorter distance to hospital.

**Source of information:** Responsible physician, medical reports.

**Comments**:

**Other quality dimension covered by this QI:** None.

**Type of quality indicator:** Process.

## Effectiveness:

### QI 11 Guidelines:

**Definition of QI:** Did the service have a guideline for the main medical problem encountered in the response?

- Yes.
- No.
- I don`t know.

**Why this QI is important:** Guidelines represent the service`s common medical practice and should be based on updated medical knowledge. Guidelines can take out some variations of different physician competence and secure that same standard of care is provided.

**Source of information:** Responsible physician. Administrative data.

**Comments**: We define a guideline as: The P-EMS` own written document that describes how to approach a medical condition or an operative situation. Both documents written as guidelines and standard operating procedures (SOP) will be included in this definition.

**Other quality dimension covered by this QI:** Equity.

**Type of quality indicator:** Structure.

### QI 12 Advanced treatment:

**Definition of QI:**

Did the P-EMS service provide advanced treatment in the actual response?

1. Yes; procedures (both medical and rescue techniques) or medications only offered by P-EMS units in the actual region.

Note: The P-EMS doctor may prior to her/his arrival, guide collaborating services to provide procedures that they otherwise don`t provide. If so, it qualifies for this response alternative.

1. Yes; procedures or medications also offered by other local pre-hospital units than the P-EMS, but these are NOT present on scene.

E.g. An EMS may have ambulances with different competences. Some ambulances may have the capacity of doing Basic Life Support (BLS), while others can perform Advanced Life Support (ALS). In a cardiac arrest response; if the present ambulance is only certified for BLS, the P-EMS` advanced life support will qualify for this answer alternative. This will be the case even when other local ambulances can perform ALS, but are not present. Moreover, this answer alternative should be used when P-EMS as the ONLY medical resource present gives treatment normally offered by ordinary EMS.

1. Yes; avoidance of unethical/unnecessary treatment

E.g. referring patients to own GP or self-help where relevant, down-scaling the level of admitting facility, avoiding futile treatment, etc.

1. Yes; presence in particularly demanding situations, e.g. the death of a child, major incidents etc. (The presence of P-EMS is considered supportive for both other care-givers and relatives).
2. No

**Why this QI is important:** Describes the added value of P-EMS involvement in the actual response.

**Source of information:** Responsible physician, medical reports.

**Comments**:

**Other quality dimension covered by this QI:** None.

**Type of quality indicator:** Process.

### QI 13 Logistics:

**Definition of QI:** Did the *logistical* contribution by P-EMS give the patient a significant better service than the existing alternative?

- 1: Yes; by reducing the estimated time to admitting facility with ≥ 30 minutes for time critical conditions like STEMI, stroke and severe trauma.
- 2: Yes; by reducing the estimated time to admitting facility with 15-29 minutes for time critical conditions like STEMI, stroke and severe trauma.
- 3: Yes; by accessing and/or evacuating the patient from an area otherwise difficult to access.

E.g.: Patients picked up from areas unaccessible by regular EMS (land and sea).

- 4: Both 1 and 3.
- 5: Both 2 and 3.
- 6: No

**Why this QI is important:** This QI illustrates when the contribution of P-EMS is to solve pure logistical challenges. Some responses performed by P-EMS is due to the patient`s remote location and/or the non-availability of ordinary EMS-units. This QI can illustrate in which regions the availability of ordinary EMS-units is poor, and for patients in these regions, the dispatch of a P-EMS unit can prevent under- treatment and secure a timely and equitable care.

**Source of information:** EMCC, medical reports, responsible physician.

**Comments**:

**Other quality dimension covered by this QI:** Timeliness. Equity.

**Type of quality indicator:** Process.

### QI 14 Research:

**Definition of QI:** Was the patient enrolled in a scientific study involving the pre-hospital care?

- Yes.
- No.
- I don`t know.

**Why this QI is important:** P-EMS should contribute to building an evidence-based practice

**Source of information:** Responsible physician, medical reports.

**Comments**:

**Other quality dimension covered by this QI:** None.

**Type of quality indicator:** Structure.

## Patient-centeredness:

### QI 15 Care for relatives:

**Definition of QI:**

Did you ensure that the relatives` needs were addressed – either by P-EMS or by collaborating services?

1. Yes; their need for transportation to admitting hospital.

(the issue can be addressed either by P-EMS, EMS or other services present on scene).

1. Yes; their need for information and emotional or social assistance.

(the issue can be addressed either by P-EMS, EMS or other services present on scene).

1. Yes; both 1 and 2.
2. No. (No time to inform relatives because of time critical situation etc.)
3. No relatives present on scene.

**Why this QI is important:** Addressing these issues is about taking care of the patient`s relatives, who are also in a stressful and difficult situation. This is important for the relatives themselves, but also for the patient in given situations. Especially if the patient or relative is a child, addressing these needs is considered particularly important.

**Source of information:** Responsible physician, medical reports.

**Comments**: Many of the patients taken care of by P-EMS are not capable of expressing their concerns and needs during the pre-hospital time interval. The care for relatives is therefore considered to be an acceptable surrogate measure for the quality dimension of patient-centeredness. Moreover, escorting relatives can have a favorable effect for paediatric patients, and even for adult patients with significant anxiety.

A relative is defined like family, friends or others who are close to the patient. Notice: Response alternative 2 above includes the notification of Child protection services when indicated.

**Other quality dimension covered by this QI:** None.

**Type of quality indicator:** Process.

# System-specific quality indicators

### QI 16 Physician-staffed Emergency Medical Communication Central (EMCC):

**Definition of QI:** Is the EMCC staffed 24/7 by specially trained pre-hospital physician?

- Yes.
- No.

**Why this QI is important:** The staffing of the EMCC by a specially trained pre-hospital physician may be beneficial for correct dispatch, major incident command and day-to-day telemedical backup/supervision of ambulances (and P-EMS). It is important that this is a 24/7 staffing in order to provide equitable care.

**Source of information:** EMCC.

**Comments**:

**Quality dimension covered by this QI:** Effectiveness (primary quality dimension), Equity, Timeliness, Safety.

**Type of quality indicator:** Structure.

### QI 17 P-EMS units per population:

**Definition of QI:** What is the number of P-EMS units per 100 000 inhabitants in the service area?

**Why this QI is important:** Gives an indication of the availability of the P-EMS service.

**Source of information:** Administrative/organizational information; annual number of citizens in area covered by the service.

**Comments**: This QI can be evaluated in relation to another QI: “P-EMS units per km²”.

**Quality dimension covered by this QI:** Equity (primary quality dimension), timeliness.

**Type of quality indicator:** Structure.

### QI 18 P-EMS units per km²:

**Definition of QI:** What is the number of P-EMS units per km^2^ in the area covered by the service?

**Why this QI is important:** Gives an indication of the availability of the P-EMS service.

**Source of information:** Administrative/organizational information; service area in km^2^.

**Comments**: This QI can be evaluated in relation to another QI: “P-EMS units per population”.

**Quality dimension covered by this QI:** Equity (primary quality dimension), timeliness.

**Type of quality indicator:** Structure.

### QI 19 Transfer and retrieval service:

**Definition of QI:** Does the service regularly perform interfacility transports/secondary transports coordinated by a dispatch centre?

- Yes.
- No.

**Why this QI is important:** Secures high quality critical care during transport with skills, knowledge and equipment tailored to the pre-hospital environment.

**Source of information:** Administrative/organizational information.

**Comments**:

**Quality dimension covered by this QI:** Effectiveness (primary quality dimension), safety.

**Type of quality indicator:** Structure.

### QI 20 Type of in-hospital service:

**Definition of QI:** What level of regular in-hospital service do the P-EMS doctors practice in addition to the pre-hospital work?

1. Level 1; i.e. university hospital/supraregional hospital.
2. Level 2; i.e. regional hospital.
3. Level 3; i.e. local hospital.
4. No in-hospital service.

**Why this QI is important:** In order to bring a high level of hospital care to the pre-hospital patients, the P-EMS doctors should work regularly in hospitals providing high level of care.

**Source of information:** Administrative/organizational information.

**Comments**: On or more response alternatives can be checked.

**Quality dimension covered by this QI:** Effectiveness.

**Type of quality indicator:** Structure.

### QI 21 Proportion of specialists:

**Definition of QI:** Proportion of doctors with achieved speciality in:

1. Anesthesiology.
2. Emergency Medicine.
3. Other (Other specialties).

**Why this QI is important:** Reflects the competence of the P-EMS-doctors. The physician must be a specialist in airway, respiratory and circulatory management. He/she operates as the only physician in an often austere environment, and must be capable to perform advanced procedures, and even guiding the treatment of other colleagues and healthcare workers.

**Source of information:** Administrative/organizational information.

**Comments**: The proportion of specialists to other doctors should be estimated with the service`s total number of P-EMS doctors as the denominator. The nominator should be the number of doctors with achieved specialty in anesthesiology, emergency medicine and other specialties, respectively.

**Quality dimension covered by this QI:** Effectiveness (primary quality dimension), equity.

**Type of quality indicator:** Structure.

### QI 22 Major incident medical management:

**Definition of QI:** Proportion of the P-EMS physicians who have attended and passed formalized training in major incident management.

Nominator: Number of P-EMS physicians who have completed a program in major incident management. Denominator: The service`s total number of P-EMS physicians.

**Why this QI is important:** To secure that the P-EMS-doctor, in collaboration with other authorities, has the ability to lead, command and control the scene in major incidents.

**Source of information:** Administrative/organizational information.

**Comments**: This QI does not refer to a specific course. Any formalized training program covering the actual topics will be satisfactory. The training should be teaching a systematic approach to disaster medical management. It should consist of both theoretical lectures and practical training.

**Quality dimension covered by this QI:** Efficiency (primary quality dimension), safety.

**Type of quality indicator:** Structure.

### QI 23 Qualification of P-EMS doctor`s assistant:

**Definition of QI:** Proportion of P-EMS physicians` assistants with the following qualification: Paramedic or nurse with:

1. Supplemental regular training in assisting during induction of general anesthesia.

AND/OR

1. Formal education in anesthesia or intensive care.

**Why this QI is important:** Those providing anesthesia should always have an anesthesia-educated assistant nearby..

**Source of information:** Administrative/organizational information.

**Comments**: Nominator: Number of P-EMS physicians` assistants with the demanded qualification (1 or/and 2). Denominator: The service`s total number of P-EMS physicians` assistants.

**Quality dimension covered by this QI:** Safety (primary quality dimension), effectiveness.

**Type of quality indicator:** Structure.

### QI 24 Patient satisfaction:

**Definition of QI:** Does the P-EMS service collect data pertaining to patient satisfaction?

- Yes; continuously.
- Yes; occasionally.
- No.

**Why this QI is important:** To evaluate the satisfaction of the P-EMS patients.

**Source of information:** Patient questionnaire/patient survey.

**Comments**:

**Quality dimension covered by this QI:** Patient-centeredness.

**Type of quality indicator:** Structure.

### QI 25 Complaints:

**Definition of QI:** What is the number of documented complaints from patients, relatives or receiving hospitals per total number of P-EMS events (ratio)?

- Nominator: Number of documented complaints for the last calendar year. Denominator: Total number of P-EMS events for the last calendar year.

**Why this QI is important:** To identify potential weak points of the P-EMS service.

**Source of information:** Administrative data.

**Comments**: We define P-EMS events as: all documented requests to P-EMS teams on call, including both counseling and completed or aborted responses.

**Quality dimension covered by this QI:** Patient-centeredness (primary quality dimension), Safety.

**Type of quality indicator:** Outcome.

### QI 26 Clinical governance:

**Definition of QI:** Does it exist a system for registration and reviewing of adverse events, critical incidents and educational events in the service?

- Yes; and the events are regularly shared in open forums or plenary meetings.
- Yes; but the events are not regularly shared in open forums or plenary meetings.
- No.

**Why this QI is important:** Audits, clinical governance, plenary meetings or other systematic feed-back loops are necessary to achieve improvement. Advanced interventions should only be performed if careful audit of activity is in place to identify quality assurance and learning objectives.

**Source of information:** Administrative data.

**Comments**: An optimal system for collecting and reviewing events/incidents, should systematically ensure that information is shared with colleagues.

**Quality dimension covered by this QI:** Safety (primary quality dimension), effectiveness.

**Type of quality indicator:** Structure.

# Key variables:

Data variable nr. 1-19 are collected from a consensus-based template for documenting and reporting in physician-staffed pre-hospital services.(8)

### Data variable no. 1:

**Data variable name:** Age

**Type of data:** Continuous

**Data variable categories or values:** Number

**Definition of data variable:** The patient`s age at the time of the event

**Comments:**

### Data variable no. 2:

**Data variable name:** Gender

**Type of data:** Nominal

**Data variable categories or values:**

1= Female

2= Male

3= Unknown

**Definition of data variable:** The patient`s gender

**Comments:**

### Data variable no. 3:

**Data variable name:** Co-morbidity

**Type of data:** Ordinal

**Data variable categories or values:**

1= No (ASA-PS = 1)

2= Yes (ASA-PS = 2-6)

2= Unknown

**Definition of data variable:**

ASA-PS definition

1= A normal healthy patient

2= A patient with mild systemic disease

3= A patient with severe systemic disease

4= A patient with severe systemic disease that is a constant threat to life

5= A moribund patient who is not expected to survive without operation

6= A declared brain-dead patient whose organs are being removed for donor purposes

**Comments:**

### Data variable no. 4:

**Data variable name:** Medical problem (main reason for response)

**Type of data:** Nominal

**Data variable categories or values:**

1= Cardiac arrest

2= Trauma

3= Breathing difficulties

4= Chest pain

5= Stroke

6= Acute neurology excluding stroke

7= Psychiatry including intoxication

8= Obstetrics and childbirth

9= Infection

10= Other

**Definition of data variable:** Select the condition most likely to be the patient`s true medical problem.

**Comments:** Defined according to EED-project and ScanDoc phase 1. The first 9 categories should include >95% of all medical conditions present in daily work.

### Data variable no. 5: (for trauma patients only)

**Data variable name:** Dominating type of injury.

**Type of data:** Nominal.

**Data variable categories or values:**

1= Blunt

2= Penetrating

3= Unknown

**Definition of data variable:** Indication of the type of injury produced if trauma

**Comments:**

### Data variable no 6:

**Data variable name:** Glascow coma scale-first

**Type of data:** Ordinal

**Data variable categories or values:** Number (3-15)

**Definition of data variable:** First recorded pre-interventional Glascow Coma Scale upon arrival of physician-staffed service.

**Comments:**

### Data variable no. 7:

**Data variable name:** Glascow coma scale-last

**Type of data:** Ordinal

**Data variable categories or values:** Number (3-15)

**Definition of data variable:** Glascow Coma Scale at end of care or patient handover.

**Comments:**

### Data variable no. 8:

**Data variable name:** Heart rate-first

**Type of data:** Continuous

**Data variable categories or values:** Number (per minute)

**Definition of data variable:** First heart rate per minute measured by physician-staffed service. If ventricular rate and pulse rate is not the same, ventricular rate should be registered.

**Comments:**

### Data variable no. 9:

**Data variable name:** Heart rate-last

**Type of data:** Continuous

**Data variable categories or values:** Number (per minute)

**Definition of data variable:** Heart rate per minute at end-of-care or patient handover

**Comments:**

### Data variable no. 10:

**Data variable name:** Systolic blood pressure-first

**Type of data:** Continuous

**Data variable categories or values:** Number (mmHg)

**Definition of data variable:** First recorded systolic blood pressured measured by physician-staffed service (measured with sphygmomanometer, monitor or intra-arterial line)

**Comments:**

### Data variable no. 11:

**Data variable name:** Systolic blood pressure-last

**Type of data:** Continuous

**Data variable categories or values:** Number (mmHg)

**Definition of data variable:** Systolic blood pressured at end-of-care or patient handover

**Comments:**

### Data variable no. 12:

**Data variable name:** Cardiac rhythm-first

**Type of data:** Ordinal

**Data variable categories or values:**

1 = Sinus rhythm

2 = SVES, VESmono.

3 = Atrial Fib/Flutt, AV-block gr. II/III, VESpoly

4 = VF, VT, Asystole, PEA

5 = Not recorded

**Definition of data variable:** First cardiac rhythm interpreted by physician-staffed service (minimum 3-channel lead)

**Comments:**

### Data variable no. 13:

**Data variable name:** Cardiac rhythm-last

**Type of data:** Ordinal

**Data variable categories or values:**

1 = Sinus rhythm

2 = SVES, VESmono.

3 = Atrial Fib/Flutt, AV-block gr. II/III, VESpoly

4 = VF, VT, Asystole, PEA

5 = Not recorded

**Definition of data variable:** Cardiac rhythm at end-of-care or patient handover

**Comments:**

### Data variable no. 14:

**Data variable name:** SpO2-first

**Type of data:** Continuous

**Data variable categories or values:** Number (0-100)

**Definition of data variable:** First recorded oxygen-saturation by physician-staffed service (measured with pulse oxymeter or arterial blood gas (SaO2))

**Comments:**

### Data variable no. 15:

**Data variable name:** SpO2-last

**Type of data:** Continuous

**Data variable categories or values:** Number (0-100)

**Definition of data variable:** Oxygen-saturation at end-of-care or patient handover

**Comments:**

### Data variable no. 16:

**Data variable name:** Pain-first

**Type of data:** Ordinal

**Data variable categories or values:**

1 = None

2 = Moderate

3 = Severe

**Definition of data variable:** First level of pain assessed by physician-staffed service

**Comments:**

### Data variable no. 17:

**Data variable name:** Pain-last

**Type of data:** Ordinal

**Data variable categories or values:**

1 = None

2 = Moderate

3 = Severe

**Definition of data variable:** Level of pain at end-of-care or patient handover

**Comments:**

### Data variable no. 18:

**Data variable name:** Respiratory rate-first

**Type of data:** Continuous

**Data variable categories or values:** Number (per minute)

**Definition of data variable:**  First respiratory rate per minute measured by physician-staffed service. If mechanically ventilated, document ventilation rate.

**Comments:**

### Data variable no. 19:

**Data variable name:** Respiratory rate-last

**Type of data:** Continuous

**Data variable categories or values:** Number (per minute)

**Definition of data variable:**  Respiratory rate at end of care or patient handover

**Comments:**

### Data variable no. 20: (only for patients who are intubated or have a supraglottic device)

**Data variable name:** EtCO2-first

**Type of data:** Continuous

**Data variable categories or values:** Number (kPa or mmHg)

**Definition of data variable:** First EtCO2 measured by physician-staffed service (for patients who are intubated or ventilated via a supraglottic device).

**Comments:**

### Data variable no. 21: (only for patients who are intubated or have a supraglottic device)

**Data variable name:** EtCO2-last.

**Type of data:** Continuous.

**Data variable categories or values:** Number (kPa or mmHg).

**Definition of data variable:** EtCO2 at end of care or patient handover (for patients who are intubated or ventilated via a supraglottic device).

**Comments:**

# References

1. Porter ME. What is value in health care? The New England journal of medicine. 2010;363(26):2477-81.

2. Institute of Medicine. Emergency Medical Services at a Crossroads. The National Academies Press, Washington DC, USA2006.

3. World Health Organization. Quality of care: a process for making strategic choices in health systems. 2006.

4. National Committee for Quality Assurance. The essential guide to health care quality. <http://www.ncqa.org/Newsroom/ResourceLibrary/EssentialGuidetoHealthCareQuality.aspx>.

5. Mainz J. Defining and classifying clinical indicators for quality improvement. International journal for quality in health care : journal of the International Society for Quality in Health Care / ISQua. 2003;15(6):523-30.

6. Institute of Medicine. Crossing the Quality Chasm: A New Health System for the Twenty-first Century. Washington: National Academies Press; 2001.

7. Lexicon: Dictionary of Health Care Terms, Organizations and Acronymes. 2nd ed: Joint Commission on Accreditation of Healthcare Organizations; 1998.

8. Kruger AJ, Lockey D, Kurola J, Di Bartolomeo S, Castren M, Mikkelsen S, et al. A consensus-based template for documenting and reporting in physician-staffed pre-hospital services. Scandinavian journal of trauma, resuscitation and emergency medicine. 2011;19:71.
