# Supplementary material for: Developing quality indicators for physician-staffed emergency medical services: a consensus process
Source: Scand J Trauma Resusc Emerg Med. 2017 Feb 15;25:14. doi: 10.1186/s13049-017-0362-4 (PMC5311851; doi:10.1186/s13049-017-0362-4)
Supplement: Additional file 1: — “Definition catalogue from the EQUIPE-project”. This explanation and elaboration document contains the definitions of all quality indicators, as well as explanation of the response alternatives where necessary. (ZIP 134 kb) [file 13049_2017_362_MOESM1_ESM.zip › Members of the EQUIPE expert panel.docx]

Members of the EQUIPE expert panel

**Dr. Gry Elise Albrektsen**

General practitioner, Florø, Norway.

**Dr. Peter Anthony Berlac, MHM**

Medical Director, Emergency Medical Services Copenhagen.

Consultant Anaesthesiologist and Prehospital Emergency Physician.

**Dr. Geir Sverre Braut**

Professor, University of Stavanger, Norway.

Former Deputy Director General, Norwegian Board of Health Supervision.

Specialist in community medicine.

**Dr. Per Bredmose**

Consultant in Prehospital Care and Retrieval Medicine, Oslo, Norway. Director of Training.

Consultant Anaesthesiologist.

**Dr. Robert Burman, PhD** (absent from the consensusmeeting)

General practitioner and Consultant, Emergency Ward, Kristiansand, Norway.

Researcher, National Centre for Emergency Primary Health Care.

**Dr. Brian Burns, Msc**

Ass. Professor of Emergency Medicine, University of Sydney.

Prehospital & Retrieval Specialist, Sydney HEMS. Ambulance Service of NSW Director of Trauma.

**Dr. Alasdair Corfield** (absent from the consensusmeeting)

Consultant in Emergency & Retrieval Medicine

Clinical Director EM (Clyde)

Honorary Clinical Associate Professor, University of Glasgow

**Dr. Marta Ebbing, PhD**

Department Director, Norwegian Institute of Public Health.

Specialist in Cardiology.

**Dr. Magnus Hjortdahl**

General practitioner, Alta, Norway.

**Dr. Freddy Lippert**

CEO Emergency Medical Services, Copenhagen.

Associate Professor, University of Copenhagen.

**Dr. Pål Madsen**

Medical advisor at Luftambulansetjenesten ANS

(Principal organization for Norwegian air ambulance services)

**Per Oretorp**

Ass. secretary general, National association for the Traumatically Injured, Norway.

**Dr. Leif Rognås, PhD** (absent from the consensusmeeting)

Lead Clinician at the Pre-hospital Critical Care Service. HEMS physician and consultant anaesthetist.

**Dr. Julian Thompson**

Consultant Anaesthesiologist og Deputy Editor Scand J Trauma Emerg Med.

**Dr. Oddvar Uleberg**

Consultant in Anaesthesiology and Pre-hospital Critical Care, Trondheim, Norway.

**Dr. Janne Virta ***

Consultant Anaesthesiologist, HEMS-doctor. Medical Advisor, FinnHEMS, Finland.

**A.o. Univ.‐Prof. Dr. Wolfgang G. Voelckel, M.Sc.**

Department of Anesthesiology and Critical Care Medicine, AUVA Trauma Center, Salzburg, Austria. Medical Director, Christophorus Flugrettung, Vienna, Austria.

**Dr. Ryan Wubben** (absent from the consensusmeeting)

Clinical Associate Professor, Department of Emergency Medicine, University of Wisconsin School of Medicine and Public Health, USA.
Medical Director and Flight Physician, UW Med Flight.

*=deceased
